# P263: Infection control-uphill task in India

**DOI:** 10.1186/2047-2994-2-S1-P263

**Published:** 2013-06-20

**Authors:** R Rao, R Mani

**Affiliations:** 1 Clinical Microbiology, Apollo Hospital, Hyderabad, India; 2 Infection Control and Prevention, Apollo Hospital, Hyderabad, India

## Introduction

In an environment of limited resources and where medical practice is biased towards intervention rather than prevention, it is not surprising that basic infection control (IC) programmes are often lacking in India. Multiple factors like lack or shortage of basic requirement (e.g gloves, hand washing facility) negligible administrative support, financial constraints, overcrowding, understaffing, low levels of staff preparedness and knowledge of key factors contribute to poor IC practices. There is wide gap between knowing and doing.

## Objectives

To have a robust, continuously active, implementable infection control and prevention (ICPC) programme. Zero tolerance to HAI and involve one and all healthcare worker.

## Methods

Started with a never give up attitude and often no member turning up for ICPC meetings. Persistence, perseverance and dedicated effort over a decade and a half has made a great impact. Now monthly ICPC meetings is attended by 30 relevant members and management agrees to what ICPC says. Continuous surveillance, analysis of data, timely IC implementation is our strength today.

## Results

Analysis of HAI surveillance data over last five years will be shared.

Our IC efforts show:

90 bedded ICU HAI in 2008 was 5.82% has been brought down to 3.25% in 2012 by active surveillance and working out IC implementable solution at once with all the stakeholders.

CLBSI, VAP and CAUTI, Device Utilization Rates are comparable to the best of the hospitals abroad. We have 4 Infection Control Nurses and conduct IC master trainer course for 200 nurses twice a year so awareness of IC practices is 90% across hospital and hand hygiene compliance 70%.Prophylactic antibiotic policy compliance is 70%.Acinetobacter infections are sporadic. Special settings like dialysis, endoscopy, long term care facility are also covered. There has been no seroconversion in our dialysis unit since last four years.

## Conclusion

Continuous and persistent effort makes the management value IC impact.

IC effectiveness in adequate resources settings such as ours is a remarkable example of the implementation of interventions to prevent and control HAI. Simple and applicable prevention measures and tools do exist- it needs the will to do by one and all in the health care system.

## Competing interests

None declared

